# Factors associated with birth satisfaction in Chinese women: a prospective cohort study

**DOI:** 10.3389/fpubh.2026.1916038

**Published:** 2026-09-09

**Authors:** Yanchi Wang, Jian Gu

**Affiliations:** 1 Affiliated Nantong Hospital of Shanghai University (The Sixth People’s Hospital of Nantong), Nantong, Jiangsu, China; 2 School of Nursing and Rehabilitation, Nantong University, Nantong, Jiangsu, China

**Keywords:** birth satisfaction, Chinese women, mode of delivery, postpartum recovery, pregnancy pressure, resilience

## Abstract

**Background:**

This study aimed to evaluate birth satisfaction and identify its key influencing factors among Chinese women, acknowledging the increasing significance of a positive birth experience in perinatal care.

**Methods:**

Between February and August 2023, 635 women were assessed during the third trimester and again at 6 weeks postpartum. Prenatal psychological and psychosocial factors were measured using validated scales, and birth satisfaction was assessed using the Chinese Birth Satisfaction Scale-Revised Indicator. Stepwise multiple linear regression was retained as an exploratory analysis, and a theory-driven hierarchical regression model was performed as a sensitivity analysis, entering mode of delivery and physical discomfort in Block 1 and prenatal psychological and psychosocial variables in Block 2.

**Results:**

Mean birth satisfaction was 8.54 (SD = 2.16). Feeding pattern and gestational weight gain were not significantly associated with birth satisfaction in the univariate analyses. The final hierarchical model explained 21.3% of the variance in birth satisfaction (*R*^2^ = 0.213, adjusted *R*^2^ = 0.204; *F* = 24.215, *p* < 0.001). Pregnancy pressure (*β* = −0.242, *p* < 0.001), mode of delivery (*β* = 0.220, *p* < 0.001), resilience (*β* = 0.147, *p* = 0.002), and physical discomfort (*β* = −0.090, *p* = 0.013) remained independently associated with birth satisfaction. In this cohort, caesarean delivery was associated with a higher birth satisfaction score than vaginal delivery; however, elective and emergency caesarean deliveries and several relevant intrapartum characteristics were not distinguished.

**Conclusion:**

Birth satisfaction among Chinese women was associated with mode of delivery, pregnancy pressure, resilience, and physical discomfort. These findings support continuous, individualized care across pregnancy, childbirth, and early postpartum recovery.

## Introduction

The subjective birth experience of the mother is a crucial aspect of obstetric care as it directly influences the health of both the mother and the newborn (1, 2). A positive childbirth experience brings lasting benefits, including a strong bond with the newborn and a positive attitude towards motherhood, which helps to enhance women’s self-esteem and sense of accomplishment (3). Conversely, a negative birth experience can lead to adverse outcomes such as postpartum depression, post-traumatic stress disorder, future fear of childbirth, and difficulties with breastfeeding adjustment (4). Therefore, optimizing the mother’s childbirth experience is essential for improving overall maternal and child health (5). Recent evidence further indicates that fear of childbirth, birth satisfaction, and postpartum depressive symptoms are interrelated and may influence early mother–infant bonding, reinforcing the clinical importance of identifying adverse childbirth experiences (6).

During labour and delivery, women attach great importance to the following aspects: their ability to give birth physiologically, their autonomy in decision-making, their sense of control over the delivery process, and the ability to achieve positive outcomes for themselves and their newborns (7). Positive childbirth experiences, as assessed by scientific evidence and emotional support, reveal the accessibility of respectful healthcare (8–11). However, the narratives discovered indicate excessive use or underutilization of intervention measures during childbirth, coupled with instances of disrespect and abusive professional conduct (9, 10). Childbirth can be unpredictable and frightening (7), which needs to be regulated by the woman’s physical and psychosocial status during pregnancy and childbirth, and by support from both family and healthcare staff in local obstetric services (7). Access to care is a key factor in achieving a positive childbirth experience, increasing women’s satisfaction with their birth and restoring confidence in healthcare professionals in obstetric care. In this context, midwives and obstetric nurses play a crucial role not only in providing support tailored to the needs of women but also in facilitating the physiological aspects of childbirth and promoting evidence-based practices (12).

WHO antenatal care guidelines integrate evidence from qualitative systematic reviews that demonstrate the significance women attach to the cultural, psychological, and emotional experiences of pregnancy, along with their own health and the wellbeing of their developing baby (13). These reviews also indicate that women perceive pregnancy, childbirth, and the postnatal period as a unified continuum of physical and mental wellbeing, rather than three distinct and unrelated states. Due to the widespread occurrence of reported negative birth experiences and their correlation with adverse outcomes, it is imperative to implement early screening and management of risk factors (14). The postpartum experience enables women to adapt to a new self-identity and develop mental resilience and capacity as mothers, to adjust to changes in intimate and family relationships, to cope with physical changes and emotional challenges, to adapt to changes in sleep patterns, to embrace the “new normal” of motherhood and parenting within their own cultural background, to receive support from family, society, and healthcare professionals, and to experience dynamic personal growth achievements (15). This connection is often described as a sense of coherence. Individuals with a strong sense of coherence are adept at utilizing existing and potential resources, have more social support, they show flexibility in their decision making, which helps them manage stress well, remain resilient and positive, and may see their lives as coherent (16). Coherence is associated with women’s birth outcomes, including birth satisfaction (17). Prenatal anxiety is associated with negative birth experiences (18). Increased communication and shared decision-making before, during and after birth may help to engage women actively in their own delivery and reduce caesarean section rates, and the mode of delivery is one aspect that affects birth satisfaction (19).

Despite a well-established body of international evidence on birth satisfaction, research within the specific cultural and healthcare context of China remains limited. Few studies have used validated, targeted measures to examine the combined contribution of sociodemographic, obstetric, psychological, psychosocial, and postpartum factors among Chinese women (20). The Birth Satisfaction Scale-Revised Indicator (BSS-RI) is a brief self-report measure of birth satisfaction comprising six items across two domains: stress experienced during childbirth and quality of care (21). The scale has been translated and validated in Chinese. Accordingly, this study aimed to measure birth satisfaction using the Chinese BSS-RI and to examine factors associated with birth satisfaction across sociodemographic characteristics, obstetric factors, prenatal psychological and psychosocial conditions, and postpartum physical discomfort. We hypothesized that birth satisfaction would be associated with mode of delivery, prenatal anxiety and pregnancy pressure, resilience, perceived social support, and postpartum physical discomfort.

The study was guided by a conceptual framework in which maternal sociodemographic and obstetric characteristics represent background conditions; perceived social support and resilience represent prenatal psychosocial resources; pregnancy pressure, anxiety, and depressive symptoms represent prenatal psychological burdens; and mode of delivery and postpartum physical discomfort represent more proximal clinical and experiential factors. Together, these domains form a multidomain maternal-care framework encompassing prenatal psychological burdens, psychosocial resources, intrapartum clinical experiences, postpartum physical recovery, and selected lifestyle-related maternal characteristics, which may jointly contribute to women’s appraisal of childbirth and subsequent birth satisfaction.

## Methods

### Participants

**Figure 1:**
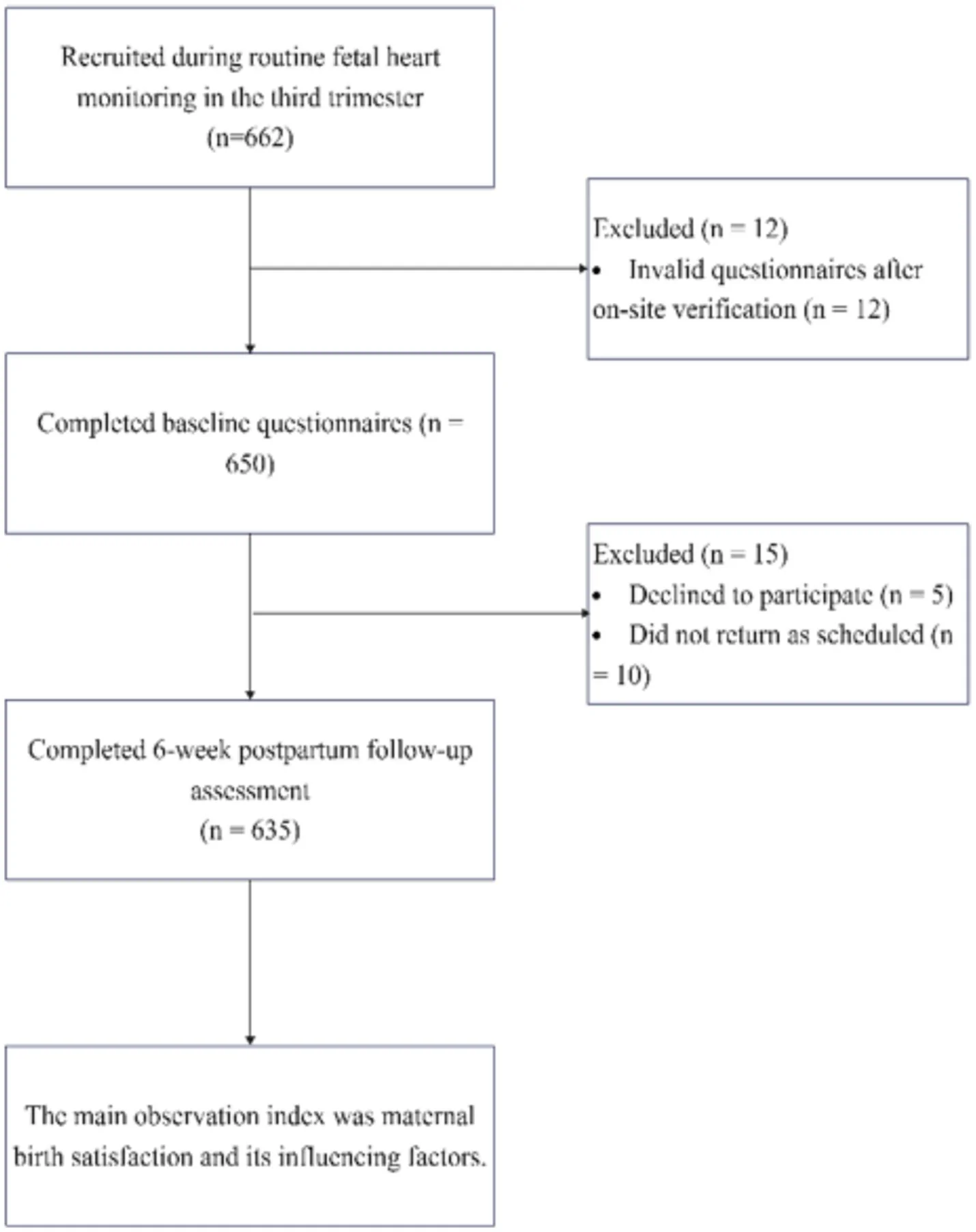
Participant recruitment and follow-up flow diagram.

This study was conducted in three tertiary-level hospitals in Nantong City, Jiangsu Province, China. These hospitals provide comprehensive maternal care services including antenatal care (ANC), intrapartum monitoring, and postnatal care. From February to August 2023, we recruited women in their third trimester of pregnancy during routine foetal heart monitoring visits using a consecutive sampling method. After a detailed explanation of the study purpose, 662 eligible women provided written informed consent and completed the baseline questionnaires. Following immediate on-site verification, 650 valid questionnaires were retained, resulting in a valid response rate of 98.2%. A dedicated database was established to track participants’ contact details and expected delivery dates through the hospital information system. For the 6-week postpartum follow-up, participants received SMS and telephone reminders to return for assessment. At this stage, 15 participants were lost to follow-up (5 declined participation, 10 did not return), resulting in a final analytical sample of 635 participants with complete data and a follow-up response rate of 97.7%. The participant recruitment and follow-up process is presented in Figure 1. All participants had experienced full-term deliveries (defined as ≥37 weeks of gestation). The sample is most representative of women without psychiatric history or severe obstetric complications receiving care at urban tertiary hospitals in China. Inclusion criteria were as follows: (1) Women aged 18 years or older, (2) In the third trimester of a singleton pregnancy, (3) Able to complete both antenatal and postpartum follow-up surveys, (4) Provided written informed consent. Exclusion criteria were as follows: (1) Known history of psychiatric disorders (e.g., diagnosed depression or anxiety disorders), (2) Major medical complications during pregnancy (e.g., pre-eclampsia, gestational diabetes requiring hospitalization), (3) Presence of malignant disease, (4) Poor literacy or cognitive impairments that made independent completion of the questionnaires unfeasible, (5) Major neonatal complications (e.g., congenital anomalies requiring immediate surgery, neonatal death, or NICU admission for more than 7 days).

A prospective power analysis was conducted using G*Power software (version 3.1.9.7) to determine the minimum sample size required for the multiple linear regression analysis. The analysis was set with the following parameters: an anticipated medium effect size (*f*^2^ = 0.15), a significance level (α) of 0.05, a statistical power (1-*β*) of 0.95, and 22 predictor variables. The calculation indicated that a minimum of 230 participants was required. To account for a potential attrition rate of approximately 20%, the target sample size was increased to 288.

### 1.1 Questionnaire survey

This was a prospective cohort study that used self-administered questionnaires to investigate the factors influencing birth satisfaction among women. The anonymous questionnaire collected data in two parts: (1) sociodemographic characteristics and related questionnaires were collected during the third trimester. The third trimester was chosen as a critical period when women are more likely to experience heightened psychological stress and physical discomfort, both of which can significantly influence their subsequent birth experience. The psychosocial state during late pregnancy is often a strong predictor of postpartum outcomes. It is recognized that emotional conditions such as stress, anxiety, or depression may change between the third trimester and the postpartum period, potentially introducing recall or measurement bias. However, the third trimester is a critical period for assessing psychological preparedness, which has been shown to influence women’s perception of birth. (2) Delivery information and The Birth Satisfaction Scale-Revised Indicator (BSS-RI), were collected at 6 weeks postpartum. The assessment of birth satisfaction at 6 weeks postpartum aligns with the standard timing of the postnatal check-up in the studied hospitals, thereby maximizing response rates and facilitating clinical relevance. Although earlier assessment might reduce recall bias, the 6-week interval represents a period when the initial intensity of postpartum recovery has stabilized, allowing women to provide a more reflective and integrated evaluation of their birth experience. To minimize recall bias and the influence of major intervening events, we used a short, focused tool (BSS-RI) and excluded women with major neonatal complications. This corresponds to a total interval of about 2 to 4 months between the two data collection points. To ensure consistency between the two questionnaire time points, women were recruited in person during routine foetal monitoring visits and asked to provide contact information. Follow-up at 6 weeks postpartum was conducted in the hospital during scheduled postnatal check-ups. Research assistants were present to clarify instructions, and participants were free to complete the forms at their own pace. Questionnaires were checked immediately after completion, and participants were invited to complete unintentionally omitted items voluntarily. Questionnaires with less than 80% completion at either assessment were considered invalid and excluded. No statistical imputation was performed, and the final analysis was based on participants with complete data for the outcome and included predictors.

### Measurement

#### Sociodemographic characteristics

Two questionnaires were designed for this study. One of the questionnaires was to collect general characteristic information of pregnant women during the third trimester. The following variables are included in the primary factors: (1) basic demographic information, including age, educational background of the pregnant woman, educational background of her husband, monthly family income, medical insurance, and place of residence, and (2) factors related to maternity, including parity, pregnancy-related complications, abortion history, assisted reproduction, and planned pregnancy. Another general questionnaire was collected at 6 weeks postpartum and included feeding patterns, mode of delivery, baby sex, birth weight of the baby, weight gain during pregnancy, physical discomfort, and breast pain. Feeding pattern was categorized as breastfeeding, mixed feeding, or formula feeding, and gestational weight gain was categorized as <10 kg, 10–15 kg, or >15 kg. These variables were collected as general maternal characteristics rather than comprehensive measures of maternal nutrition. Detailed dietary intake, maternal nutritional status, nutrition counselling, breastfeeding exclusivity over time, and breastfeeding duration were not assessed. In our study, physical discomfort was assessed based on participants’ self-reported symptoms during the postpartum period, including perineal pain, incisional pain, and general musculoskeletal discomfort. Breast pain was defined based on the clinical criteria commonly used in obstetric and postpartum care, including tenderness, swelling, or pain localized in the breast tissue during the postpartum period. This includes pain caused by engorgement due to delayed breastfeeding, poor latch, or milk stasis. The classification was based on patient self-report in combination with standard postpartum nursing documentation. Mode of delivery was recorded as either vaginal delivery or caesarean section; elective or planned and emergency or unplanned caesarean sections were not recorded separately.

In the participating hospitals, neuraxial labour analgesia was available to eligible women following maternal request and obstetric and anaesthetic assessment. Routine non-pharmacological comfort measures included breathing guidance, position adjustment, relaxation techniques, and support from obstetric nurses or midwives. Nevertheless, individual-level information regarding the use, timing, effectiveness, accessibility, or reasons for the non-use of labour analgesia was not collected in this study.

### Questionnaires collected during the third trimester

#### Pregnancy pressure scale (PPS)

The PPS, developed by Chen et al. (22), was a self-report assessment tool for evaluating stress during pregnancy, which takes into account the cultural framework of China in its design. The PPS consists of 30 items, and each item is rated on a 4-point scale (0 = not at all, 1 = mild, 2 = moderate, 3 = severe). The total score is calculated by dividing the actual score by the number of items, and a higher total score indicates a higher level of stress experienced during pregnancy (23). In the present study, the Cronbach’s α for the PPS was 0.942.

#### Depression anxiety stress scale-21 (DASS-21)

The DASS-21 was a tool used to assess the level of negative emotions and severity of symptoms experienced by individuals in the previous week (24). The scale consists of 21 items, including three sub-scales: depression, anxiety, and stress, with each subscale comprising 7 questions. Each item is rated on a 4-point scale (0 = completely inconsistent, 1 = partially consistent, 2 = mostly consistent, 3 = completely consistent). The total score of this scale is calculated by multiplying the raw score by two, and the higher the total score, the stronger the negative emotions are represented. The DASS-21 has been demonstrated to be cross-culturally valid in mainland China and the Cronbach’s α of 0.83, 0.80, and 0.82 for the Depression, Anxiety, and Stress subscales, respectively, and 0.92 for the total DASS total (25). In this study, the internal consistency for the anxiety subscale of the DASS-21 was good, with a Cronbach’s α of 0.727.

#### The Edinburgh postnatal depression scale (EPDS)

The EPDS, developed by Cox (26), was used to assess the self-reported symptoms of depression. The Chinese version of this scale has been validated by Wang et al. and can be used to screen for postpartum depression (27). The scale consists of 10 items, with scores ranging from 0 to 3 for each item. The total score ranges from 0 to 30, with higher scores indicating a greater likelihood of depression. In China, a cut-off point of 10 points is used to assess symptoms of postpartum depression. The Cronbach’s α for the EPDS in this sample was 0.852, indicating good reliability.

#### 10-item Connor–Davidson resilience scale (CD-RISC-10)

The CD-RISC-10 scale, developed by Connor and Davidson (28) was used in this study in its Chinese version translated and revised by Chinese researchers. It consists of 10 items, and each item is rated using a 5-point Likert scale. A score of 0 represents “never,” 1 represents “rarely,” 2 represents “sometimes,” 3 represents “often,” and 4 represents “always.” The scores of all items are summed to obtain a total score, which ranges from 0 to 40. A higher score indicates a higher level of resilience. The translated version of the scale demonstrated good internal consistency with a Cronbach’s alpha coefficient of 0.92 (29). This scale is also applicable to pregnant women in China (30). In our sample, the scale demonstrated excellent internal consistency, with a Cronbach’s α of 0.946.

#### Perceived social support scale (PSSS)

The Chinese version of PSSS was developed by Zimet (31), and is used to assess the perceived level of social support. The scale consists of 12 items, divided into three subscales: family support (4 items), friend support (4 items), and others support (4 items). Each item is rated on a 7-point Likert scale, ranging from 1 (strongly disagree) to 7 (strongly agree). A higher total score indicates a stronger perception of social support (32). The scale demonstrates good internal consistency, with a Cronbach’s alpha coefficient of 0.88. The Cronbach’s α for the PSSS in this study was 0.948, indicating excellent reliability.

#### Questionnaires collected at 6 weeks postpartum

##### The Birth Satisfaction Scale-Revised Indicator (BSS-RI)

BSS-RI (21) was a brief measurement tool that has been proven to be reliable and valid for self-reporting birth satisfaction. It consists of 6 items, divided into 2 sub-scales: stress experienced during childbearing (BSS-RI-SE) with 4 items, and quality of care (BSS-RI-QC) with 2 items. Each item was scored on a 3-point Likert scale (0 = disagree, 1 = somewhat agree, 2 = agree). Items 1, 3, and 6 were positively scored, while items 2, 4, and 5 were negatively scored. Scores range from 0 to 12, with higher total scores indicating higher birth satisfaction. This researcher has translated and verified the Chinese version of BSS-RI (33). The CFA showed good model fit with χ^2^/Df = 1.511, RMSEA = 0.035, CFI = 0.989, TLI = 0.979, and NFI = 0.968. In this study, the Cronbach’s α for the BSS-RI was 0.755. The primary outcome measure was the total score on the Chinese version of the Birth Satisfaction Scale-Revised Indicator (C-BSS-RI).

### Data analysis

SPSS 25.0 (IBM Corp., Armonk, NY) was used for statistical analysis. Descriptive statistics were used to summarize participant characteristics. Independent-samples *t*-tests and one-way analysis of variance were used to compare BSS-RI scores across categorical variables, and Pearson correlation analysis was used for continuous variables. Stepwise multiple linear regression, with the total BSS-RI score as the dependent variable, was retained as an exploratory analysis because the relative independent contributions of the candidate psychosocial and clinical factors had not been fully established. Variables showing statistically significant group differences or correlations with the BSS-RI score were entered as candidate predictors. As a theoretically informed sensitivity analysis, hierarchical multiple linear regression was additionally performed. Mode of delivery and physical discomfort were entered in Block 1, followed by pregnancy pressure, resilience, prenatal anxiety, perceived social support, and prenatal depression in Block 2. Changes in model explanatory ability were evaluated using Δ*R*^2^ and the *F*-change test. Before the final model was interpreted, the assumptions of multiple linear regression were examined. Normality was assessed using a histogram and normal probability plot of standardized residuals. Linearity and homoscedasticity were evaluated using a scatterplot of standardized residuals against standardized predicted values. Independence of residuals was assessed using the Durbin–Watson statistic. Potentially influential observations were examined using standardized residuals, leverage values, and Cook’s distance. Multicollinearity was assessed using tolerance and variance inflation factors. Potential interaction effects between key variables were explored by including interaction terms; none were statistically significant. Statistical significance was set at *p* < 0.05 (two-tailed).

### Ethical consideration

This study was reviewed and approved by the Institutional Ethics Committee (approval number: 2022-K150-01). This study adheres to guidelines to ensure voluntary and confidential participation. Written informed consent was obtained from all participants and required to sign an informed consent form prior to data collection.

## Results

### Participants’ demographic characteristics

A total of 635 mothers completed questionnaires at two time points: the third trimester and 6 weeks postpartum. Birth satisfaction scores did not differ significantly based on age, mother’s education, family monthly income, medical insurance, place of residence, mode of feeding, baby sex, baby weight, pregnancy weight gain, pregnancy complications, parity, abortion history, assisted reproduction, and planned pregnancy (*p* ≥ 0.05). Specifically, birth satisfaction did not differ significantly across feeding patterns (*F* = 0.962, *p* = 0.383) or gestational weight-gain categories (*F* = 1.114, *p* = 0.329). There were statistically significant differences in mode of delivery (*p* < 0.001), physical discomfort (*p* < 0.001), and breast pain (*p* = 0.020). (Table 1).

**Table 1:** Characteristics information of participants.

Variables		Scores of BSS-RI		*F/t*	*P*	Variables		Scores of BSS-RI		*F/t*	*P*
		N (%)	Mean± SD					N (%)	Mean ± SD		
Age	<30	316(49.8%)	8.43 ± 2.18	−1.295	0.196	Baby sex	Boy	326 (51.3%)	8.63 ± 2.00	1.040	0.299
	≥30	319 (50.2%)	8.66 ± 2.13				Girl	309 (48.7%)	8.45 ± 2.31		
Education (oneself)	Below bachelor	224 (35.3%)	8.64 ± 2.26	0.845	0.399	Abortion history	No	455 (71.7%)	8.49 ± 2.15	−0.977	0.329
	Bachelor or above	411 (64.7%)	8.49 ± 2.10				Yes	180 (28.3%)	8.68 ± 2.17		
Mode of delivery	Vaginal delivery	412 (64.9%)	8.17 ± 2.13	−6.124	<0.001	Planned pregnancy	No	96 (15.1%)	8.56 ± 1.87	−0.097	0.923
	Caesarean section	223 (35.1%)	9.24 ± 2.03				Yes	539 (84.9%)	8.54 ± 2.21		
Monthly family income (CNY ¥)	<5,000	19 (3.0%)	7.89 ± 2.42	1.705	0.165	Parity	Primipara	486 (76.5%)	8.47 ± 2.15	−1.557	0.120
	5,000–10,000	195 (30.7%)	8.75 ± 2.11				Multipara	149 (23.5%)	8.79 ± 2.17		
	10,001–20,000	302 (47.6%)	8.41 ± 2.14			Birth weigh	<3 kg	87 (13.7%)	8.66 ± 2.34	0.312	0.732
	>20,000	119 (18.7%)	8.66 ± 2.23				3–4 kg	484 (76.2%)	8.55 ± 2.10		
Medical insurance	No	45 (7.1%)	8.38 ± 2.28	−0.539	0.590		>4 kg	64 (10.1%)	8.38 ± 2.33		
	Yes	590 (92.9%)	8.56 ± 2.15			Complications of pregnancy	No	450 (70.9%)	8.46 ± 2.21	−1.654	0.099
Place of residence	Urban	391 (61.6%)	8.55 ± 2.20	0.175	0.840		Yes	185 (29.1%)	8.76 ± 2.01		
	Towns	169 (26.6%)	8.48 ± 2.08			Assisted reproduction	No	516 (81.3%)	8.51 ± 2.14	0.903	0.367
	Rural	75 (11.8%)	8.65 ± 2.14				Yes	119 (18.7%)	8.71 ± 2.23		
Mode of feeding	Breast-feeding	224 (35.3%)	8.71 ± 2.16	0.962	0.383	Pregnancy weight gain	<10 kg	114 (18.0%)	8.82 ± 2.00	1.114	0.329
	Mixed-feeding	312 (49.1%)	8.45 ± 2.12				10-15 kg	341 (53.7%)	8.50 ± 2.10		
	Formula-feeding	99 (15.6%)	8.47 ± 2.27				>15 kg	180 (28.3%)	8.46 ± 2.35		
Breast pain	No	567 (89.3%)	8.61 ± 2.16	2.332	0.020	Physical discomfort	No	367 (57.8%)	8.80 ± 2.13	3.573	<0.001
	Yes	68 (10.7%)	7.97 ± 2.02				Yes	268 (42.2%)	8.19 ± 2.15		

#### Mean, standard deviation (SD), and correlations for study variables

The scores for several scales were as follows: birth satisfaction was 8.54 (SD = 2.16), social support was 72.30 (SD = 9.75), resilience was 28.21(SD = 6.49), pregnancy pressure was 0.41(SD = 0.37), prenatal anxiety was 5.15 (SD = 4.57), and prenatal depression was 5.15(SD = 4.31). As summarized in Table 2, birth satisfaction was significantly positively correlated with social support (*r* = 0.237, *p* < 0.001) and resilience (*r* = 0.290, *p* < 0.001). Conversely, it was negatively correlated with pregnancy pressure (*r* = −0.362, *p* < 0.001), prenatal anxiety (*r* = −0.238, *p* < 0.001), and prenatal depression (*r* = −0.258, *p* < 0.001).

**Table 2:** Mean, standard deviation (SD), and correlations for study variables (*N* = 635).

Variables	Birth satisfaction	Social support	Resilience	Pregnancy pressure	Prenatal anxiety	Prenatal depression
Birth satisfaction	1					
Social support	0.237^***^	1				
Resilience	0.290^***^	0.623^***^	1			
Pregnancy pressure	−0.362^***^	−0.351^***^	−0.410^***^	1		
Prenatal anxiety	−0.238^***^	−0.278^***^	−0.345^***^	0.522^***^	1	
Prenatal depression	−0.258^***^	−0.501^***^	−0.545^***^	0.548^***^	0.535^***^	1
Min	2	35	10	0	0	0
Max	12	84	40	1.93	28	19
Mean	8.54	72.30	28.21	0.41	5.15	5.15
Standard deviation (SD)	2.16	9.75	6.49	0.37	4.57	4.31

***p < 0.001 (two-tailed test).

### Exploratory stepwise regression

Exploratory stepwise regression (Table 3, Panel A) retained four variables independently associated with birth satisfaction: mode of delivery, pregnancy pressure, resilience, and physical discomfort. The final model explained 21.0% of the variance in birth satisfaction (*R*^2^ = 0.210, adjusted *R*^2^ = 0.205; *F* (4, 630) = 41.816, *p* < 0.001). Greater pregnancy pressure (*β* = −0.260, *p* < 0.001) and physical discomfort (*β* = −0.094, *p* = 0.009) were associated with lower birth satisfaction, whereas greater resilience (*β* = 0.175, *p* < 0.001) and caesarean delivery, compared with vaginal delivery (*β* = 0.219, *p* < 0.001), were associated with higher birth satisfaction.

**Table 3:** Exploratory stepwise and hierarchical multiple linear regression analyses of factors associated with the C-BSS-RI total score (*N* = 635).

Variables		B	SE	*β*	95% CI		*P*	*VIF*
					Lower	Upper		
Panel A. Exploratory stepwise regression								
BSS-RI	PPS	−1.523	0.231	−0.260	−1.978	−1.069	<0.001	1.240
	CD-RISC-10	0.058	0.013	0.175	0.033	0.084	<0.001	1.203
	Physical discomfort	−0.409	0.157	−0.094	−0.717	−0.100	0.009	1.031
	Mode of delivery	0.988	0.160	0.219	0.673	1.303	<0.001	1.006

Variables	*B*	SE	*β*	95%CI		*t*	*P*	VIF
				Lower	Upper			
Panel B. Hierarchical regression sensitivity analysis								
(Constant)	7.068	0.782	-	5.532	8.603	9.038	<0.001	-
Block 1: Clinical factors								
Physical discomfort	−0.392	0.158	−0.090	−0.703	−0.082	−2.485	0.013	1.043
Mode of delivery	0.992	0.161	0.220	0.677	1.307	6.179	<0.001	1.007
Block 2: Psychological and psychosocial factors								
Pregnancy pressure	−1.420	0.267	−0.242	−1.945	−0.895	−5.308	<0.001	1.656
Prenatal anxiety	−0.018	0.021	−0.038	−0.059	0.023	−0.858	0.391	1.576
Prenatal depression	0.010	0.025	0.020	−0.040	0.060	0.386	0.699	2.040
Social support	0.013	0.010	0.059	−0.006	0.032	1.310	0.191	1.602
Resilience	0.049	0.016	0.147	0.018	0.079	3.146	0.002	1.739

PPS, Pregnancy Pressure Scale.

CD-RISC-10: 10-item Connor–Davidson Resilience Scale.

Model summary: *R*^2^ = 0.210; adjusted *R*^2^ = 0.205; *F* (4,630) = 41.816, *p* < 0.001; standard error of the estimate = 1.923; Durbin–Watson = 1.903.

Block 1 *R*^2^ = 0.078. The addition of psychological and psychosocial variables in Block 2 increased the explained variance by 13.5% (Δ*R*^2^ = 0.135, *p* < 0.001). Final model: *R*^2^ = 0.213, adjusted *R*^2^ = 0.204, *F* = 24.215, *p* < 0.001; standard error of the estimate = 1.923; Durbin–Watson = 1.903.

### Hierarchical regression sensitivity analysis

In the hierarchical regression sensitivity analysis (Table 3, Panel B), mode of delivery and physical discomfort entered in Block 1 explained 7.8% of the variance in birth satisfaction (*R*^2^ = 0.078). The addition of psychological and psychosocial variables in Block 2 significantly increased the explained variance by 13.5% (Δ*R*^2^ = 0.135, *p* < 0.001). The final model explained 21.3% of the variance in birth satisfaction (*R*^2^ = 0.213, adjusted *R*^2^ = 0.204; *F* = 24.215, *p* < 0.001). Pregnancy pressure (*β* = −0.242, *p* < 0.001), mode of delivery (*β* = 0.220, *p* < 0.001), resilience (*β* = 0.147, *p* = 0.002), and physical discomfort (*β* = −0.090, *p* = 0.013) remained independently associated with birth satisfaction. Prenatal anxiety, prenatal depression, and perceived social support were not independently associated with birth satisfaction in the fully adjusted model (all *p* > 0.05).

Residual diagnostics confirmed that the residuals were approximately normally distributed, and no systematic pattern was observed between residuals and predicted values, supporting the assumptions of normality, linearity, and homoscedasticity. The Durbin–Watson statistic was 1.903. All Cook’s distance values were below 1, and no observation showed an excessively high leverage value. VIF values ranged from 1.007 to 2.040, indicating no problematic multicollinearity.

## Discussion

This prospective cohort study examined factors associated with birth satisfaction among Chinese women. Mean birth satisfaction was 8.54 ± 2.16. The exploratory stepwise model and theory-driven hierarchical sensitivity analysis produced consistent findings: mode of delivery, pregnancy pressure, resilience, and physical discomfort remained independently associated with birth satisfaction, whereas prenatal anxiety, prenatal depression, and perceived social support were not independently associated after simultaneous adjustment. These findings support a multifactorial interpretation in which prenatal psychosocial resources and burdens, together with more proximal clinical and postpartum experiences, jointly contribute to women’s birth satisfaction.

Previous studies have reported inconsistent associations between mode of delivery and birth satisfaction. Some studies have suggested that women undergoing caesarean delivery may report greater satisfaction because of better psychological preparation, reduced labour pain, and a stronger sense of predictability or control (19, 34, 35). In contrast, lower satisfaction after caesarean delivery has also been reported, particularly when the procedure is unplanned or differs from women’s prenatal expectations (36). Other studies have found no clear difference in satisfaction between vaginal and caesarean delivery (37, 38). These inconsistent findings indicate that birth satisfaction may be influenced not only by the delivery route itself, but also by maternal expectations, perceived control, communication with healthcare professionals, clinical circumstances, and the quality of intrapartum care.

In the present study, women who underwent caesarean delivery reported higher birth satisfaction than those who had a vaginal delivery. However, this association should be interpreted cautiously and should not be regarded as evidence that caesarean delivery itself produces a better childbirth experience. In the context of urban tertiary hospitals in China, some women may place considerable importance on clinical safety, predictability, access to medical technology, and avoidance of unexpected complications. Recent evidence among Chinese pregnant women showed that intolerance of uncertainty was associated with caesarean-section preference through fear of childbirth, suggesting that predictability and psychological preparation may influence women’s delivery preferences (39). By contrast, prolonged labour, severe pain, perineal injury, unexpected interventions, or a perceived loss of control may negatively influence women’s evaluations of vaginal birth (40). Satisfaction may therefore depend partly on whether the actual birth experience is consistent with a woman’s expectations and whether she receives adequate information, emotional support, pain relief, and respectful care. Because elective and emergency caesarean deliveries, prenatal delivery preferences, clinical indications, labour duration, and analgesia use were not separately assessed, the observed association remains cohort-specific and may be affected by residual confounding.

Pain management may also partly explain the difference in satisfaction between delivery modes. Women undergoing vaginal delivery may experience pain over a longer and less predictable period, whereas anaesthesia is routinely provided during caesarean delivery. Differences in the availability, timing, effectiveness, and perceived control of pain relief may therefore influence women’s overall appraisal of childbirth. Prospective evidence comparing water immersion with epidural analgesia also indicates that different pain-management pathways may shape maternal satisfaction with the childbirth experience (41). As individual-level information on labour analgesia was not collected, the contribution of pain-management practices could not be examined directly and should be investigated in future studies.

Higher resilience was independently associated with greater birth satisfaction. Previous studies have shown that resilience is positively related to psychological wellbeing and adaptive coping (42, 43). Childbirth may be experienced as a major physical and emotional challenge, and women with greater resilience may be better able to regulate negative emotions, mobilize coping resources, and adapt to uncertainty, pain, and changes in perceived control. Antenatal interventions such as emotion-regulation training, cognitive and behavioural strategies, physical activity, sleep support, mindfulness, and individualized childbirth preparation may help strengthen resilience (44). Nevertheless, because this was an observational study, the findings indicate an association rather than a causal effect of resilience on birth satisfaction.

Greater pregnancy pressure was independently associated with lower birth satisfaction. The Pregnancy Pressure Scale reflects concerns about maternal and fetal safety, adaptation to the parental role, and changes in body shape and physical activity (23). These concerns may be particularly prominent among first-time mothers who lack previous childbirth experience and feel uncertain about labour, infant health, personal safety, and the transition to motherhood. Adequate prenatal preparation may reduce uncertainty and help women develop more realistic expectations of childbirth, thereby contributing to a more satisfactory birth experience (45). Healthcare professionals should therefore identify pregnancy-related pressure during routine antenatal care and provide clear information, shared decision-making, individualized psychological support, and practical preparation for labour and postpartum recovery.

Although perceived social support was positively correlated with birth satisfaction in the univariate analysis, it was not independently associated with birth satisfaction after simultaneous adjustment for clinical and psychosocial factors. Perceived social support was strongly correlated with resilience in this study, suggesting that their associations with birth satisfaction may partly reflect shared variance. Conceptually, social support may strengthen women’s coping resources and resilience or reduce pregnancy-related pressure. However, social support, resilience, and pregnancy pressure were measured at the same antenatal assessment, and no formal mediation analysis was prespecified. Therefore, the present findings do not establish that resilience mediates the relationship between social support and birth satisfaction. Longitudinal studies with repeated measurements and prespecified mediation models are needed to examine these potential pathways (46).

Postpartum physical discomfort was independently associated with lower birth satisfaction. Perineal pain, incisional pain, musculoskeletal discomfort, breast engorgement, and breastfeeding-related pain may influence how women retrospectively appraise childbirth. Timely pain assessment, individualized pharmacological and non-pharmacological management, perineal and wound care, breastfeeding support, and clear communication during early recovery may help reduce discomfort and support a more positive integrated appraisal of childbirth.

Birth satisfaction assessed at 6 weeks postpartum may reflect not only the childbirth event itself but also women’s subsequent interpretation of that event during early motherhood. Breastfeeding difficulties, postpartum mood symptoms, infant health concerns, sleep disruption, family support, and the course of physical recovery may alter the salience or meaning of the original birth experience. The outcome in this study should therefore be understood as a six-week retrospective and integrated appraisal of childbirth rather than an immediate post-delivery assessment.

From a multidomain maternal-care perspective, women’s appraisal of childbirth may reflect prenatal psychological burdens, psychosocial resources, intrapartum clinical experiences, postpartum physical recovery, and selected lifestyle-related maternal characteristics. Feeding pattern and gestational weight gain were examined in this study, but neither was significantly associated with birth satisfaction. These findings should be interpreted cautiously because detailed dietary behaviour, maternal nutritional status, nutrition support, breastfeeding exclusivity, and breastfeeding duration were not assessed.

This study has several strengths, including its prospective design, large sample, high follow-up rate, and use of validated measures. Several limitations should nevertheless be considered. First, birth satisfaction was assessed at 6 weeks postpartum. Although this timing corresponds to routine postnatal care and may allow a more reflective evaluation, it increases the possibility of recall bias and influence from intervening experiences, including breastfeeding, postpartum mood, infant health, sleep disruption, family support, and physical recovery. Future studies should assess satisfaction both shortly after delivery and at subsequent postpartum follow-up. Second, the DASS-21 anxiety subscale reflects symptoms during the preceding week; consequently, the antenatal assessment may not represent emotional status during labour. Third, mode of delivery was recorded only as vaginal delivery or caesarean section. Elective and emergency caesarean deliveries were not distinguished, although these subtypes may differ in expectations, perceived control, clinical urgency, psychological preparation, and satisfaction. Fourth, several established determinants of birth satisfaction were not measured, including the availability, use, and effectiveness of labour analgesia; prenatal birth expectations and concordance between preferred and actual delivery; respectful maternity care; continuity of midwifery care; the presence and role of a birth companion; induction and duration of labour; instrumental delivery; intrapartum complications; and indication for caesarean section. Although women whose neonates required more than 7 days of intensive care were excluded, short-term neonatal observation or intensive care was not separately examined. These unmeasured characteristics may have produced residual confounding, particularly in the association between mode of delivery and birth satisfaction. Fifth, participants were consecutively recruited from three tertiary hospitals in a single city, and women with pre-existing psychiatric conditions or major obstetric or neonatal complications were excluded. The sample may therefore have been healthier than the general obstetric population, potentially overestimating satisfaction and limiting external validity. Women receiving care in primary or secondary facilities, rural regions, or different healthcare systems may have different expectations and clinical experiences. In addition, feeding pattern and gestational weight gain were assessed using broad categories, while detailed dietary behaviour, maternal nutritional status, nutrition counselling, breastfeeding exclusivity, and breastfeeding duration were not collected. Therefore, the study cannot clarify nutrition-related mechanisms or evaluate the effects of nutrition support on birth satisfaction. Finally, the psychosocial variables were measured at the same antenatal time point, precluding conclusions about temporal or mediating pathways. Future multicentre longitudinal studies should include repeated psychosocial assessments, detailed intrapartum variables, and prespecified mediation or structural equation models.

## Conclusion

Birth satisfaction was independently associated with mode of delivery, pregnancy pressure, resilience, and physical discomfort. In this cohort, caesarean delivery was associated with a higher satisfaction score, but this cohort-specific association should not be interpreted causally because caesarean subtypes and several relevant intrapartum factors were not measured. Continuous, individualized care should include early identification of pregnancy-related pressure, support for coping and resilience, shared decision-making, effective pain management, and timely management of postpartum discomfort.

## Data Availability

The raw data supporting the conclusions of this article will be made available by the authors, without undue reservation.
